# The Unicellular, Parasitic Fungi, Sanchytriomycota, Possess a DNA Sequence Possibly Encoding a Long Tubulin Polymerization Promoting Protein (TPPP) but Not a Fungal-Type One

**DOI:** 10.3390/microorganisms11082029

**Published:** 2023-08-07

**Authors:** Ferenc Orosz

**Affiliations:** Institute of Enzymology, Research Centre for Natural Sciences, 1117 Budapest, Hungary; orosz.ferenc@ttk.hu

**Keywords:** *Amoeboradix gromovi*, ciliome, flagellum, fungi, p25alpha domain, pseudocilium, *Sanchytrium tribonematis*

## Abstract

The unicellular, parasitic fungi of the phylum Sanchytriomycota (sanchytrids) were discovered a few years ago. These unusual chytrid-like fungi parasitize algae. The zoospores of the species of the phylum contain an extremely long kinetosome composed of microtubular singlets or doublets and a non-motile pseudocilium (i.e., a reduced posterior flagellum). Fungi provide an ideal opportunity to test and confirm the correlation between the occurrence of flagellar proteins (the ciliome) and that of the eukaryotic cilium/flagellum since the flagellum occurs in the early-branching phyla and not in terrestrial fungi. Tubulin polymerization promoting protein (TPPP)-like proteins, which contain a p25alpha domain, were also suggested to belong to the ciliome and are present in flagellated fungi. Although sanchytrids have lost many of the flagellar proteins, here it is shown that they possess a DNA sequence possibly encoding long (animal-type) TPPP, but not the fungal-type one characteristic of chytrid fungi. Phylogenetic analysis of p25alpha domains placed sanchytrids into a sister position to Blastocladiomycota, similarly to species phylogeny, with maximal support.

## 1. Introduction

The loss of the flagellum is an important step in the evolution of terrestrial fungi. How many times this loss has occurred during evolution is somewhat disputed [1,2]. The exact number also depends on which species are classified as fungi. The number of events within the Holomycota clade seems to be reaching a resting point. The loss of flagellum is accepted in amoeboid nucleariids, Microsporidia and terrestrial fungi [3]. A fourth independent loss event occurred in *Hyaloraphidium curvatum* (Monoblepharidomycota) [4]. According to the classical classification, flagellated fungi all belonged to the Chytridiomycota; this view changed in 2006, when the Blastocladiomycota were also defined as a phylum [5], then Neocallimastigomycota [6] and the early-branching Cryptomycota [7] (later named as Rozellomycota [8]), which includes the non-flagellated Microsporidia [9], became independent phyla. The classification of Tedersoo et al. [8] removed additional groups from the Chytridiomycota and defined the Monoblepharomycota and Olpidiomycota as independent phyla. Apart from these, aphelids [10] were classified as a new phylum among fungi as Aphelidiomycota [8]. It was logical since aphelids branched off after Rozellomycota (Cryptomycota) and was sister to all other fungi in their analysis. (This finding was supported by others [11].) Finally (at least until now), the phylum Sanchytriomycota (sanchytrids) was established as sister to Blastocladiomycota [3]. It should be noted that this classification (i.e., eight phyla for flagellated fungi) is not yet generally accepted.

**Figure 1:**
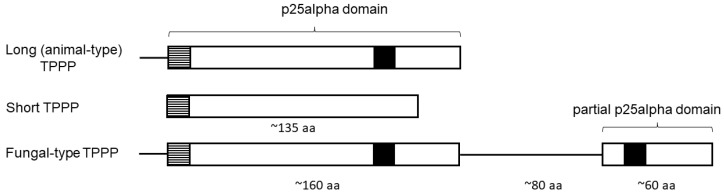
Schematic structure of long (animal-type), short and fungal-type TPPPs. Highly conservative sequence motives are denoted with black (GxGxGxxGR) and striped boxes (L(V)xxxF(Y)xxFxxF). aa—amino acid.

The loss of the flagellum is usually accompanied by the complete or the partial loss of genes/proteins related to flagellar function [3,12,13,14]. The collection of genes/proteins that are present only and exclusively in organisms with flagella or cilia (they are practically the same organelle) composes the ciliome [15]. Genes of the ciliome are generally absent in species without cilium/flagellum [15]. Tubulin polymerization promoting protein (TPPP)-like proteins seem to be part of the ciliome [12,16,17,18,19,20]. In a few cases, their role in the formation of flagellum was proven experimentally [18,19,20]. Members of this family stabilize microtubules and are characterized by the presence of the p25alpha domain(s) (Pfam05517 or IPR008907) [17] that starts generally with a L(V)xxxF(Y)xxFxxF sequence. The C-terminal part of the domain contains a very characteristic ‘Rossman-like’ sequence, GxGxGxxGR (Figure 1). These proteins can be grouped on the basis of the length and completeness of the p25alpha domain (long, short, truncated) and the presence of another kind of domain(s) [17]. (For example, apicortins contain a partial (C-terminal) p25alpha domain and a doublecortin (DCX) domain [21].) ‘Long’ TPPP (Figure 1) is present in Opisthokonta (animals, flagellated fungi, and Choanoflagellata [12,17]), and contains a ‘long’ (full length) p25alpha domain. There is only long TPPP in animals thus long TPPP is also named ‘animal-type’ TPPP [12,22]. A special, ‘fungal-type’ TPPP, which contains both a full and a partial p25alpha domain (Figure 1), is present only in certain fungi [12,22,23]. In general, there is high homology between the C-terminus of the full-length domain and the partial domain [23].

It was shown earlier that members of all phyla of flagellated fungi contain TPPP-like proteins: Rozellomycota, Chytridiomycota, Neocallimastigomycota, Monoblepharomycota, and Blastocladiomycota [12]; Olpidiomycota [23]; Aphelidiomycota [22]. On the other hand, terrestrial fungi do not contain these kinds of proteins [12]. However, it was an open question whether the members of the recently defined Sanchytriomycota phylum possess TPPP-like (p25alpha domain containing) proteins.

## 2. Methods

### 2.1. Database Homology Search

A database homology search was carried out with an NCBI Blast search [24] (http://www.ncbi.nlm. nih.gov/BLAST/, accessed on 15 May 2023): sequences of various fungal proteins containing p25alpha domain (e.g., *Batrachochytrium dendrobatidis* XP_006680205, *Chytriomyces confervae* TPX65513, TPX78276, *Neocallimastix californiae* ORY36261, *Spizellomyces punctatus* XP_016604112, XP_016606225) were used as queries against protein and nucleotide databases to find similar sequences in Sanchytriomycota (Sanchytriaceae) using BLASTP and TBLASTN analysis. The accession numbers of sequences refer to the NCBI GenBank database. The recent phylogenetic classification by Tedersoo et al. [8] was followed.

### 2.2. Phylogenetic Analysis

Multiple alignments of sequences were conducted by the Clustal Omega program [25]. Bayesian analysis, using MrBayes v3.1.2 [26], was also performed to construct a phylogenetic tree using whole sequences of TPPP proteins. Default priors and the WAG model [27] were used, assuming equal rates across sites. Two independent analyses were run with three heated and one cold chain (temperature parameter 0.2) for 4.0 × 10^−6^ generations, with a sampling frequency of 0.01, and the first 25% of generations were discarded as burn-in. The two runs were convergent.

### 2.3. Prediction of Unstructured Regions

Sequences were submitted to the IUPRED3 server freely available at http://iupred3.elte.hu/ (accessed on 6 July 2023) [28]. It was used in ‘long disorder’ mode with medium smoothing.

## 3. Results

**Figure 2:**
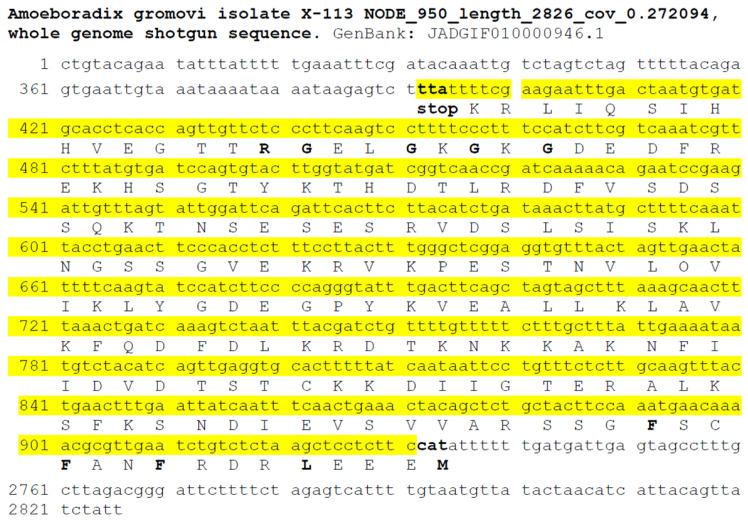
Suggested sequence of *Amoeboradix gromovi* TPPP. Numbers indicate the order of nucleotides in JADGIF010000946.1 of whole genome shotgun sequences of *A*. *gromovi*. Yellow background indicates the manually translated nucleotide sequence. The corresponding amino acids are shown with capital letters.

**Figure 3:**
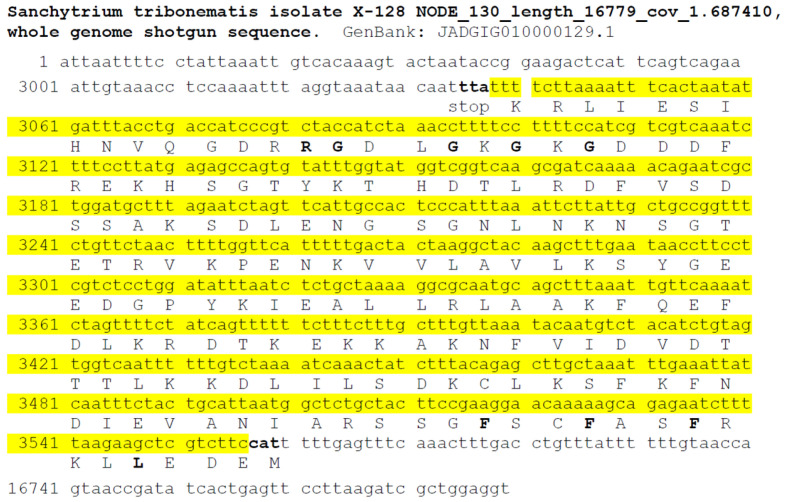
Suggested sequence of *Sanchytrium tribonematis* TPPP. Numbers indicate the order of nucleotides in JADGIG010000129.1 of whole genome shotgun sequences of *S. tribonematis.* Yellow background indicates the manually translated nucleotide sequence. The corresponding amino acids are shown with capital letters.

**Figure 4:**
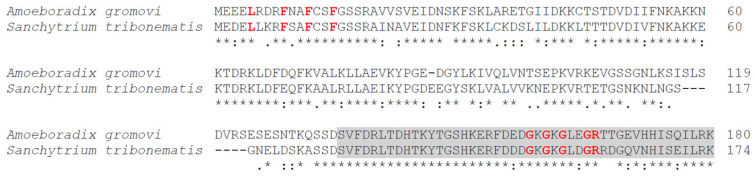
Alignment of *Amoeboradix gromovi* and *Sanchytrium tribonematis* TPPPs performed by Clustal Omega program [25]. Highly conservative sequence motives are highlighted with red bold letters: L(V)xxxF(Y)xxFxxF and GxGxGxxGR (cf. Figure 1). Identical and biochemically similar amino acids are labeled by asterisk and colon, respectively. Grey background indicates the conservative sequence of ‘partial p25alpha domain’ which is also present in apicortin and in duplicate in fungal-type TPPPs.

**Figure 5:**
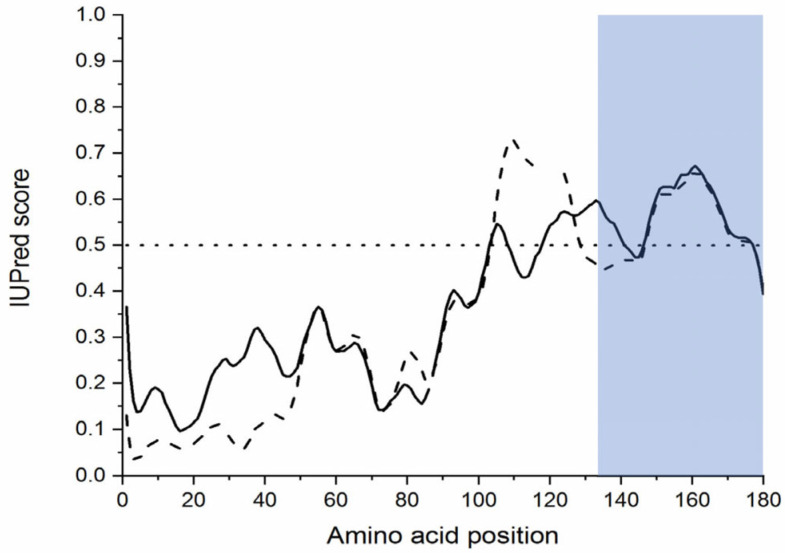
Disorder prediction of TPPPs of sanchytrids using the IUPRED3 predictor. Disorder prediction values for the given residues are plotted against the amino acid residue number; *Amoeboradix gromovi* TPPP (solid line), *Sanchytrium tribonematis* TPPP (dashed line). The significance threshold, above which a residue is considered to be disordered, set to 0.5, is shown. The C-terminal parts of the proteins corresponding to the partial p25alpha domain are indicated by a blue box.

Protein and nucleotide sequences available at the NCBI website, including transcriptome shotgun assemblies (TSAs), whole-genome shotgun contigs (WGSs), and expressed sequence tags (ESTs), were searched for the p25alpha domain containing sequences in Sanchytriomycota. Sequences of various p25alpha domain containing proteins were used as queries in the BLAST searches (cf. Methods). There were no protein hits, but such nucleotides were found in *Amoeboradix gromovi* and *Sanchytrium tribonematis* as WGS sequences, namely, in *A. gromovi* JADGIF010000946 and in *S. tribonematis* JADGIG010000129. The manual translation of these sequences indicated that *S. tribonematis* and *A. gromovi* possibly contain a *long (animal-type)* TPPP of 174 and 180 amino acid length, respectively (Figure 2, Figure 3 and Figure 4). Both TPPPs contain the motifs characteristic for p25alpha domains; e.g., L(V)xxxF(Y)xxFxxF at the very beginning of the domain and GxGxGxxGR in the C-terminal part (Figure 4). Structurally, the first half of these proteins is predicted to be ordered and the second half, which includes the sequence corresponding to the partial p25alpha domain, to be disordered (Figure 5).

No other type of protein/transcript containing p25alpha domain (short TPPP, fungal-type TPPP, apicortin) or DNA sequences encoding them was found in the BLAST search. The two nucleotide sequences found were used as queries in the BLASTX search to find the most similar proteins in the protein databases. (More precisely, only the part of the sequences corresponding to the manually translated proteins was used.) The hits with E-value smaller than 1 × 10^−30^ are listed in Table 1 and Table 2. The results indicated that these sequences are of fungal and animal origin. The best hits were long (animal-type) TPPPs (i.e., TPPPs containing a full length p25alpha domain) of animal origin. There are some fungal-type TPPPs (i.e., TPPPs containing a full length and a partial p25alpha domains) among the hits; the lowest E-value among fungal-type TPPPs was obtained for a *Paraphysoderma sedebokerense* (phylum Blastocladiomycota) protein (KAI9140125). Long TPPPs of fungal origin were represented only by *Amoeboaphelidium protococcarum* (Aphelidiomycota). Interestingly, a special protein, not known before, containing two full length p25alpha domains was also obtained as one of the best hits (KAJ3407993, *Chytridiales* sp.; phylum Chytridiomycota). 

**Figure 6:**
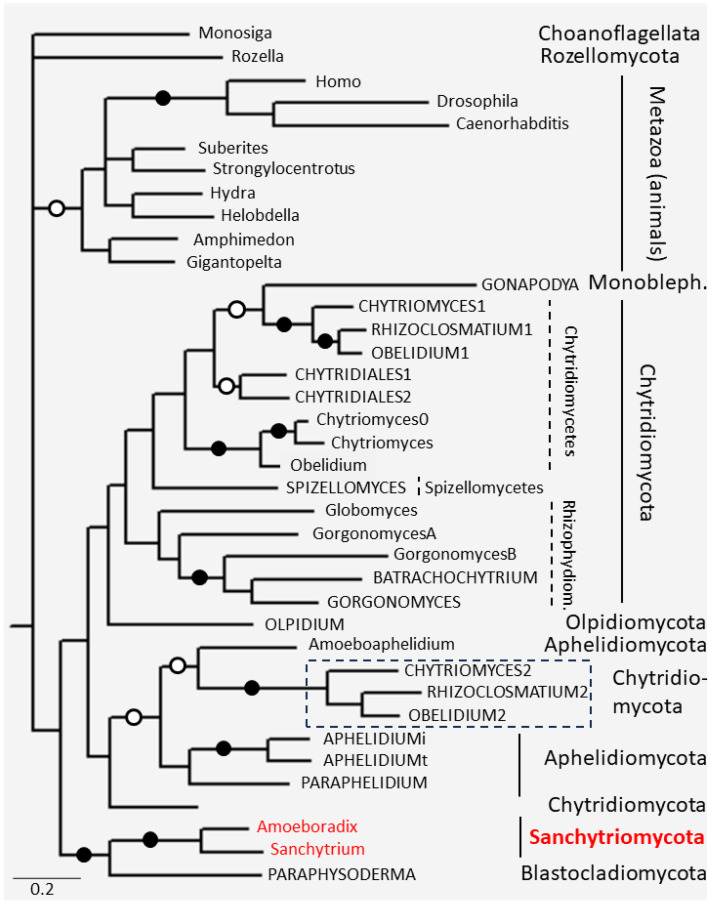
The phylogenetic tree of full length p25alpha domains. Filled and open circles at a node indicate that the branch was supported by the maximal Bayesian posterior probability (BPP) and ≥0.95 BPP, respectively. All the other branches were supported by BPP ≥ 0.5. p25alpha domain of *Monosiga brevicollis* XP_001743131 was used as outgroup. Species names with capital letters indicates fungal-type TPPPs. The box with dotted lines includes fungal-type TPPP paralogs being present only in some Chytridiomycota. Monobleph.—Monoblepharomycota; Rhizophydiom.—Rhizophydiomycetes. The accession numbers of proteins are listed in Table 1, Table 2, and Table S1.

A phylogenetic tree of p25alpha domains was constructed (Figure 6). Full-length p25alpha domains of long- and fungal-type TPPPs were used. Since the Sanchytriomycota TPPPs belong to long (animal-type) TPPPs, thus long TPPPs of Fungi were involved from all the species containing it. Domains of the most similar animal long TPPPs (cf. Table 1 and Table 2) and that of long TPPPs of some reference genomes were also included. It should be noted that in the case of long TPPPs, the p25alpha domain represents almost the whole sequence of the protein (cf. Figure 1). Full p25alpha domains of selected fungal-type TPPPs were also included from all the phyla which contain them (Aphelidiomycota, Blastocladiomycota, Chytridiomycota, and Olpidiomycota.) Both full-length 25alpha domains of the TPPP of the above-mentioned *Chytridiales* species were used in the phylogenetic analysis.

The p25alpha domains of animals and fungi form separate clades (Figure 6). The animal clade is supported by high Bayesian posterior probability (BPP). Within fungi, there are two clades: Sanchytriomycota + Blastocladiomycota, supported by maximal BPP, and all the other fungi (except the earliest branching Rozellomycota). In the former clade, Sanchytriomycota, supported by maximal BPP, is a sister to Blastocladiomycota. In the latter clade, there are two sister clades; one of them contains most of the Chytridiomycota (and the only Monoblepharomycota) in a sister position to Olpidiomycota; the other one contains Aphelidiomycota and a few Chytridiomycota. Within Chytridiomycota, the classes (Chytridiomycetes, Spizellomycetes, Rhizophydiomycetes) are well separated. It can be seen on the tree that belonging to certain phyla or classes “overwrites” whether the p25alpha domain originates from long (animal-type) or fungal-type TPPP. Long- and fungal-type TPPPs are always sisters to each other within a given phylogenetic unit. This finding is held in the case of Chytridiomycetes, Rhizophydiomycetes, and Aphelidiomycota as well.

## 4. Discussion

*S. tribonematis* [29,30,31] and *A. gromovi* [29,30] are closely related strains of chytrid-like parasites of the green-yellow alga, *Tribonema gayanum*. They are the only known members of the newly established phylum, Sanchytriomycota [3] and they are endowed with unusual features. From our point of view, the nature and the structure of their flagellum are the most interesting. Early-branching fungi, in general, reproduce by using motile flagellated zoospores. However, posterior flagellum of amoeboid zoospores of sanchytrids drags behind the cell without being involved in active locomotion, thus it can be considered as a pseudocilium. It contains a long kinetosome (basal body) composed of microtubular singlets, and the two orthogonal centrioles in their sporangia have nine microtubular singlets instead of the canonical kinetosome with nine microtubule triplets [30,31].

Sanchytrids lack several flagellar components, such as axonemal dyneins, and almost all the intraflagellar transport proteins [3]. Their kinetosomes also lost several components of the centriolar structure, as well as Delta and Epsilon tubulins, which are essential for centriolar microtubule assembly [3]. These losses explain why sanchytrids lack motile flagella. Concerning the presence/absence of flagellar components, sanchytrids are at an intermediate position between flagellated and non-flagellated lineages. Thus, according to the opinion of Galindo et al. [3], sanchytrids are in an unfinished process of flagellum loss.

However, it should be noted that sanchytrids are not alone in this special position. As mentioned, both species of the phylum parasitize the green-yellow alga, *T. gayanum*. The very same alga has other fungal parasites, *Aphelidium tribonematis* [32] and *Paraphelidium tribonematis* [33], which belong to the phylum Aphelidiomycota and did not lose their flagellum [34]. Based on the genomic/proteomic data published in references [11] and [35], it has been shown that both of them possess a fungal-type TPPP (Figure S1 and [22]). However, species of another genus of the phylum, *Amoeboaphelidium*, are characterized by the presence of a non-motile pseudocilium [10,36], similarly to sanchytrids. The presence of flagellar proteins was systematically investigated in two species, *Amoeboaphelidium protococcorum* and *Amoeboaphelidium occidentale*, where genomic/proteomic data are available [13]. Many, but not all, of the flagellar proteins were lost, which fact parallels the sanchytrids. *A. occidentale* does not have a TPPP, but *A. protococcorum* does have a long (animal-type) TPPP. It seems that the occurrence of a pseudocilium is connected to the presence of the long, but not the fungal-type TPPP (Table 3). One can speculate whether this is by chance or for some reason. The fungal-type TPPP differs from the long one in that it contains the C-terminal part of the p25alpha domain twice. This partial p25alpha domain is responsible for the tubulin/microtubule binding ability of TPPPs in animals from sponges to mammals [37,38,39]. This may be due to the fact that this part of the domain is intrinsically disordered (unstructured) (Figure 5) (i.e., so called IDP [40] or IUP [41]).

All phyla of flagellated fungi contain TPPP-like proteins, however, their distribution varies with the phyla (Table 3). Chytridiomycota is the only phylum where both long (animal-type) and fungal-type TPPPs and apicortin are present. Fungal-type TPPP, which occurs only in fungi, can be found in five out of the eight phyla. Both long TPPP and apicortin are present in four phyla. In general, it is true that all species of flagellated fungi possess at least one TPPP-like (p25alpha domain-containing) protein. The only exception is *Orpinomyces* sp. (phylum Neocallimastigomycota); other members of this phylum contain an apicortin. *Orpinomyces* sp. strain C1A was fully sequenced (estimated sequence completion was 94.4% [42]) but no TPPP-like protein was found by BLAST search. There are some ongoing sequencing projects of the species of this genus (https://mycocosm.jgi.doe.gov/pages/fungi-1000-projects.jsf) (accessed on 6 July 2023); their completion will clarify the reason of this hiatus.

Species from only two genera of the phylum Monoblepharomycota were fully sequenced; *H. curvatum* has no flagellum [4], thus, not surprisingly, lacks p25alpha domain-containing proteins. Species of the *Gonapodya* genus possess fungal-type TPPP and apicortin.

At the time of its discovery, *S. tribonematis* was classified as a Monoblepharidomycetes [29], and soon after, together with *A. gromovi*, sanchytrids were defined as a new fungal lineage which remains *incertate sedis* within fungi [30]. Finally, phylogenomic analyses by Galindo et al. [3] revealed that Sanchytriomycota form a well-supported, sister clade to Blastocladiomycota. The phylogenetic analysis of p25alpha domains of TPPPs fits to this view; Sanchytriomycota is a sister to Blastocladiomycota, supported by maximal BPP (Figure 6). Beside this fact, the phylogenetic tree confirms that fungal-type TPPP is a fungal innovation; the clade of fungal proteins is well separated from those of choanoflagellates and animals, which do not have this type of protein. An interesting point is that not all Chytridiomycota TPPP/p25alpha domain can be found in the same clade. The unusual (different from the species phylogeny) position of *Caulochytrium* TPPP was found earlier, too [22,23]; the reason for it is not known. Another case is the species included in the dotted box in Figure 6 labeled with ‘2’ following the species name. These TPPPs are so-called ‘outparalogs’ [43], which are present in the same species (e.g., *Chytriomyces confervare* or *Rhizoclosmatium globosum*) but the duplication event occurred earlier than the species speciation. They are grouped with Aphelidiomycota, in accordance with previous results [22].

Why do sanchytrids (and some Aphelidiomycota) retain a non-motile flagellum? The answer by Gallino et al. [3] is: “Since the primary flagellar function has been lost in favor of the amoeboid movement, other selective forces must be acting to retain this atypical structure for a different function in zoospores.” Their hypothesis is that the new function may be a sensory one, more precisely, the reduced flagellum could be involved in a phototactic response [3]. The sensory function seems to be a logical suggestion since the non-motile ‘sensory cilium’ is well known in animals and specialized versions of non-motile cilium are involved in many aspects of sensation [44]. The single photoreceptor sensory cilium (PSC) or outer segment elaborated by each rod and cone photoreceptor cell of the retina is a classic example [45,46], where the presence of a long (animal-type) TPPP was shown [47].

Fungi consist of 18 phyla according to the latest classification by Tedersoo et al. [8]. The 19th is Sanchytriomycota. Among these, the eight early-branching clades are the non-terrestrial fungi, which reproduce by using flagellated zoospores. In terrestrial fungi, the flagellum is lost. Thus, fungi provide an ideal opportunity to test and confirm the correlation between the occurrence of flagellar proteins (the ciliome), including very probably TPPP-like proteins, and that of the eukaryotic cilium/flagellum since the flagellum occurs in some phyla and not in others. If the flagellum has been lost for a long time (e.g., terrestrial fungi), these proteins cannot be found in the genome/proteome, not even in traces, in contrast to the situation in the flagellated species. Sometimes, they were preserved as ‘relics’ in species at smaller phylogenetic distances (e.g., in the case of the green algae genus, *Ostreococcus*, which—unlike other green algae, such as *Chlamydomonas*—lost its flagellum but contains a highly divergent TPPP ortholog [16]), in which case they may acquire a new function. Sanchytrids may be a nice example of this phenomenon.

## 5. Conclusions

Fungi provide an ideal opportunity to test and confirm the correlation between the occurrence of flagellar proteins and that of the eukaryotic cilium/flagellum since the flagellum occurs in the early-branching phyla and not in terrestrial fungi. TPPP-like proteins, which contain a p25alpha domain, also were suggested to belong to flagellar proteins [16]. *S. tribonematis* [29,30,31] and *A. gromovi* [29,30] are the only known members of the newly established fungal phylum, Sanchytriomycota [3]. The zoospores of the species of the phylum contain a non-motile pseudocilium (i.e., a reduced posterior flagellum). Although sanchytrids lost many of the flagellar proteins, here it has been shown that they possess a DNA sequence possibly encoding a long (animal-type) TPPP but not the fungal-type one characteristic for chytrid fungi. Phylogenetic analysis of p25alpha domains placed sanchytrids into a sister position to Blastocladiomycota, similarly to species phylogeny, with maximal support.

## Figures and Tables

**Table 1:** Best protein hits containing p25alpha domain when using *Amoeboradix gromovi* JADGIF010000946.1 as a query in BLASTX search on NCBI protein database.

Scientific Name ^1^	Accession No.	Phylum	E-Value ^2^	Query Cover	Identity	Length ^3^
*Suberites domuncula*	ADX30619	Porifera	2 × 10^−39^	96%	47.43%	180
*Hydra vulgaris*	XP_047138925	Cnidaria	2 × 10^−36^	87%	49.06%	167
*Amphimedon queenslandica*	XP_003384590	Porifera	2 × 10^−35^	90%	47.56%	183
*Chytridiales* sp. JEL 0842	KAJ3407993	Chytridiomycota	3 × 10^−34^	93%	45.35%	507
*Lytechinus variegatus*	XP_041483006	Echinodermata	3 × 10^−34^	91%	44.58%	171
*Strongylocentrotus purpuratus*	XP_782492	Echinodermata	7 × 10^−34^	90%	46.34%	171
*Acanthaster planci*	XP_022082363	Echinodermata	1 × 10^−33^	91%	45.51%	172
*Xenia* sp. Carnegie-2017	XP_046842992	Cnidaria	1 × 10^−33^	90%	44.24%	171
*Paraphysoderma sedebokerense*	KAI9140125	Blastocladiomycota	2 × 10^−33^	92%	44.13%	330
*Stylophora pistillata*	XP_022794224	Cnidaria	2 × 10^−33^	91%	45.78%	172
*Exaiptasia diaphana*	XP_020906468	Cnidaria	4 × 10^−33^	91%	45.18%	172
*Amoeboaphelidium protococcarum*	KAI3639621	Aphelidiomycota	8 × 10^−33^	97%	42.94%	190
*A. protococcarum*	KAI3650757	Aphelidiomycota	3 × 10^−32^	97%	42.94%	190
*Orbicella faveolata*	XP_020610915	Cnidaria	3 × 10^−32^	91%	45.51%	172
*A. protococcarum*	KAI3631655 ^4^	Aphelidiomycota	4 × 10^−32^	97%	42.94%	190
*Batrachochytrium dendrobatidis*	OAJ42615	Chytridiomycota	6 × 10^−32^	98%	41.58%	258
*B. dendrobatidis*	XP_006680205 ^4^	Chytridiomycota	2 × 10^−31^	92%	43.50%	289
*B. dendrobatidis*	OAJ42613	Chytridiomycota	2 × 10^−31^	92%	43.50%	299
*Acropora millepora*	XP_029200582	Cnidaria	2 × 10^−31^	91%	44.58%	172
*Acropora digitifera*	XP_015755004	Cnidaria	4 × 10^−31^	91%	44.58%	172
*A. protococcarum*	KAI3652328	Aphelidiomycota	5 × 10^−31^	97%	42.37%	190
*Anneissia japonica*	XP_033097468	Echinodermata	1 × 10^−30^	91%	43.37%	171
*Dendronephthya gigantea*	XP_028409959	Cnidaria	1 × 10^−30^	90%	46.95%	205
*Paramuricea clavata*	CAB4022691	Cnidaria	2 × 10^−30^	90%	46.67%	171
*Lamellibrachia satsuma*	KAI0228059	Annelida	9 × 10^−30^	90%	42.59%	160

^1^ Yellow and green background indicate fungal and animal species, respectively. Blue background indicates fungal-type TPPPs. ^2^ E-value is the measure of likeliness that sequence similarity is not by random chance. An E-value smaller than 1 × 10^−50^ includes database matches of very high quality. Blast hits with E-value smaller than 1 × 10^−2^ can still be considered as good hit for homology matches. ^3^ Magenta background indicates a protein that contains two full-length p25alpha domains. All other data apply to long (animal-type) TPPPs. ^4^ Used for phylogenetic analysis (Figure 6).

**Table 2:** Best protein hits containing p25alpha domain when using *Sanchytrium tribonematis* JADGIG010000129.1 as a query in BLASTX search on NCBI protein database.

Scientific Name ^1^	Accession No.	Phylum	E-Value ^2^	Query Cover	Identity	Length ^3^
*Hydra vulgaris*	XP_047138925	Cnidaria	9 × 10^−41^	88%	53.25%	167
*Suberites domuncula*	ADX30619	Porifera	7 × 10^−39^	95%	48.21%	180
*Amphimedon queenslandica*	XP_003384590	Porifera	6 × 10^−38^	87%	50.66%	183
*Lamellibrachia satsuma*	KAI0228059	Annelida	5 × 10^−36^	88%	46.10%	160
*Paraphysoderma sedebokerense*	KAI9140125	Blastocladiomycota	8 × 10^−35^	94%	41.81%	330
*Xenia* sp. Carnegie-2017	XP_046842992	Cnidaria	2 × 10^−34^	91%	45.68%	171
*Helobdella robusta*	XP_009017134	Annelida	1 × 10^−33^	88%	45.57%	160
*Lytechinus pictus*	XP_054770705	Echinodermata	1 × 10^−33^	93%	47.56%	170
*Capitella teleta*	ELU16892	Annelida	2 × 10^−33^	90%	47.50%	169
*Batrachochytrium dendrobatidis*	KAJ8323001	Chytridiomycota	6 × 10^−33^	98%	41.53%	258
*Paramuricea clavata*	CAB4022691	Cnidaria	1 × 10^−32^	91%	48.12%	171
*Strongylocentrotus purpuratus*	XP_782492	Echinodermata	1 × 10^−32^	91%	45.62%	171
*Lytechinus variegatus*	XP_041483006	Echinodermata	1 × 10^−32^	93%	46.34%	171
*B. dendrobatidis*	XP_006680205	Chytridiomycota	2 × 10^−32^	98%	41.53%	289
*B. dendrobatidis*	OAJ42613	Chytridiomycota	2 × 10^−32^	98%	41.53%	299
*Gigantopelta aegis*	XP_041378691	Mollusca	3 × 10^−32^	89%	44.87%	182
*Dendronephthya gigantea*	XP_028409959	Cnidaria	3 × 10^−32^	88%	49.68%	205
*Acanthaster planci*	XP_022082363	Echinodermata	7 × 10^−32^	91%	45.62%	172
*Exaiptasia diaphana*	XP_020906468	Cnidaria	7 × 10^−32^	91%	45.00%	172
*Amoeboaphelidium protococcarum*	KAI3639621	Aphelidiomycota	1 × 10^−31^	85%	44.97%	190
*Chytridiales* sp. JEL 0842	KAJ3407993	Chytridiomycota	2 × 10^−31^	98%	42.61%	507
*Orbicella faveolata*	XP_020610915	Cnidaria	5 × 10^−31^	91%	44.38%	172
*Hydractinia symbiolongicarpus*	XP_057291302	Cnidaria	1 × 10^−30^	93%	45.83%	164
*Clydaea vesicula*	KAJ3223392	Chytridiomycota	5 × 10^−30^	98%	42.39%	314
*Stylophora pistillata*	XP_022794224	Cnidaria	7 × 10^−30^	93%	45.12%	172
*Gigantopelta aegis*	XP_041378692	Mollusca	8 × 10^−30^	87%	46.41%	163
*A. protococcarum*	KAI3650757	Aphelidiomycota	9 × 10^−30^	85%	44.30%	190

^1^ Yellow and green background indicate fungal and animal species, respectively. Blue background indicates fungal-type TPPPs. ^2^ E-value is the measure of likeliness that sequence similarity is not by random chance. An E-value smaller than 1 × 10^−50^ includes database matches of very high quality. Blast hits with E-value smaller than 1 × 10^−2^ can still be considered as good hit for homology matches. ^3^ Magenta background indicates a protein that contains two full-length p25alpha domains. All other data apply to long (animal-type) TPPPs.

**Table 3:** Connection between TPPP-like proteins and flagellum in early branching fungi.

Phylum/Genus (Species)	TPPP-Like Protein			Flagellum
	Long TPPP (Animal Type)	Fungal-Type TPPP	Apicortin	
Rozellomycota	Yes	No	Yes	Yes
Aphelidiomycota	Yes	Yes	No	Yes
*Aphelidium*	No	Yes	No	Yes
*Paraphelidium*	No	Yes	No	Yes
*Amoeboaphelidium protococcorum*	Yes	No	No	Pseudocilium
*Amoeboaphelidium occidentale*	No	No	No	Pseudocilium
Neocallimastigomycota	No	No	Yes	Yes
*Orpinomyces* sp.	No	No	No	Yes
Monoblepharomycota	No	Yes	Yes	Yes
*Gonapodya*	No	Yes ^1^	Yes	Yes
*Hyaloraphidium curvatum*	No	No	No	No
Chytridiomycota	Yes	Yes	Yes	Yes
Olpidiomycota	No	Yes	No	Yes
Blastocladiomycota	No	Yes	No	Yes
Sanchytriomycota	Yes	No	No	Pseudocilium

^1^ Data have not published yet. *Gonapodya* sp. JEL0774, KAJ3339789.1. https://www.ncbi.nlm.nih.gov/protein/KAJ3339789.1. accessed on 4 July 2023).

## Data Availability

The data presented in this study are available in this paper and in the Supplementary Materials.

## Supplementary Materials

The following supporting information can be downloaded at https://www.mdpi.com/article/10.3390/microorganisms11082029/s1; Table S1: Accession numbers of proteins shown in Figure 6 not shown in Table 1 and Table 2. Figure S1: Sequences of *Aphelidium* TPPPs.
