# Supplementary material for: The Unicellular, Parasitic Fungi, Sanchytriomycota, Possess a DNA Sequence Possibly Encoding a Long Tubulin Polymerization Promoting Protein (TPPP) but Not a Fungal-Type One
Source: Microorganisms. 2023 Aug 7;11(8):2029. doi: 10.3390/microorganisms11082029 (PMC10459994; doi:10.3390/microorganisms11082029)
Supplement: Supplementary file 1 [file microorganisms-11-02029-s001.zip › microorganisms-2532444-supplementary.pdf]

Table S1. Accession Numbers of proteins shown in Figure 6[1]

Name on the Figure	Species	Phylum	NCBI Accession No.
Monosiga	*Monosiga brevicollis*	Choanoflagellata[2]	XP_001743131
Homo	*Homo sapiens*	Chordata	NP_057048
Drosophyla	*Drosophila melanogaster*	Arthropoda	NP_648881
Caernohabditis	*Caenorhabditis elegans*	Nematoda	NP_491219
ROZELLA	*Rozella allomycis*	Rozellomycota	EPZ30999
GONAPODYA	*Gonapodya* sp. JEL0774	Monoblepharomycota	KAJ3339789
Chytriomyces	*Chytriomyces* sp. MP71	Chytridiomycota	KAI8613955
Chytriomyces0	*Chytriomyces confervae*	Chytridiomycota	TPX78276
CHYTRIOMYCES1	*C. confervae*	Chytridiomycota	TPX65886
CHYTRIOMYCES2	*C. confervae*	Chytridiomycota	TPX72533
Obelidium	*Obelidium mucronatum*	Chytridiomycota	KAI9342551
OBELIDIUM1	*O. mucronatum*	Chytridiomycota	KAI9351240
OBELIDIUM2	*O. mucronatum*	Chytridiomycota	KAI9342285
RHIZOCLOSMATIUM1	*Rhizoclosmatium globosum*	Chytridiomycota	KAJ3297182
RHIZOCLOSMATIUM2	*R. globosum*	Chytridiomycota	ORY45507
SPIZELLOMYCES	*Spizellomyces punctatus*	Chytridiomycota	XP_016604112
GORGONOMYCES	*Gorgonomyces haynaldii*	Chytridiomycota	KAI8912588
GorgonomycesA	*G. haynaldii*	Chytridiomycota	KAI8912823
GorgonomycesB	*G. haynaldii*	Chytridiomycota	KAI8906053
Globomyces	*Globomyces pollinis-pini*	Chytridiomycota	KAI8895260
CAULOCHYTRIUM	*Caulochytrium protostelioides*	Chytridiomycota	RKP02545
OLPIDIUM	*Olpidium bornovanus*	Olpidiomycota	KAG5460860+<br>KAG5458366
APHELIDIUMi	*Aphelidium insulamus*	Aphelidiomycota	Cf. Fig. S2
APHELIDIUMt	*Aphelidium tribonematis*	Aphelidiomycota	Cf. Fig. S2
PARAPHELIDIUM	*Paraphelidium tribonematis*	Aphelidiomycota	TRINITY_DN24782[3]

[1]Accession Numbers listed in Table 1 and Table 2 are not shown here. [2]Class. [3]https://doi.org/10.6084/m9.figshare.7339469.v1 (accessed on 14 November 2022).

Aphelidium insulamus

>O14_transcripts_NODE_20210.p3 type: complete length:159
gc: universal O14_transcripts_NODE_20210:67-543(+)

MSLAHAFESFATFGAPNTQGPVTMDNAHFAKLCRDAHIVDKRVTAVDVDITFKQVLTKGSRRITFDQFQQGLQTLATKK
YAAAQLSDEEALKRITALVVNAAPTSSGTTADTAGIFSKLTDASQYTGSHRSRFDENGRRRYIYSCTAPCAYMLTVMCH
*

>O14_transcripts_NODE_21726.p1 type: complete length:292
gc: universal O14_transcripts_NODE_21726:67-942(+)

MS**LAHAFESFATFGAPNTQGPVTMDNAHFAKLCRDAHIVDKRVTAVDVDITFKQVLTKGSRRITFDQFQQGLQTLATKK**
YAAAQLSDEEALKRITALVVNAAPTSSGTTADTAGIFSKLTDASQYTGSHRSRFDENGNGLGLAGRDTHTRTANLSQMV
DRSIAKVPAPTSTGKRTVTLSMEEMNHQKPSSSGRRVVKPASTTPKKGPTSIGARGTVSASAQSLAKPRQANVTGSSTS
MAAKSVSGSG**SVYDRLTDSKGYTGTHKHRFDDTGKGRGMLGRD**HPTTSQILRST

Aphelidium tribonematis

>P2_transcripts_NODE_21635.p1 type:5prime_partial length:297
gc: universal P2_transcripts_NODE_21635:956-66(-)

FSPKSKLHKLIMS**LSHAFEAFATFGAPSTQGPVTMDNAHFAKLCRDARIVDKRVTSVDVDITFKQVLTKGARRITDAQF**
QQALKVLAAKKYGSNVSEADALQKISALVTQAAPVSNGTAADASGIFAKLTDTTKYTGAHRSRFDENGNGLGLAGRETH
VRTANLSDMVDRSLSKPAAPQKRTVTLSMEEMNQQQQRSSAGQRVVKPASTPKKNAAFAAKSKVGSSSQSLAKPRQTNV
SASANSLSAKSQGGSG**NVYDRLTDSKQYTGTHKHRFDDTGKGRGLAGRD**QPTSSQILRS*

>P2_transcripts_NODE_23454.p1 type:3prime_partial length:275
gc: universal P2_transcripts_NODE_23454:823-2(-)

MS**LAHAFESFATFGAPNTQGPVTMDNAHFAKLCRDAHIVDKRVTAVDVDITFKQVLTKGSRRITFDQFQQGLQTLATKK**
YAAAQLSDEEALKRITALVVNAAPTSSGTTADTAGIFSKLTDASQYTGSHRSRFDENGNGLGLAGRDTHTRTANLSQMV
DRSIAKVPAPTSTGKRTVTLSMEEMNHQKPASSGRRVVKPASTTPKKGPTSIGARGTVSASAQSLAKPRQANVTGSSTS
MAAKSVSGSGS**VYDRLTDSKGYTGTHKHRFDDTGKGR**

Figure S1. Sequences of *Ahelidium* TPPPs. The sequences were retrieved from https://figshare.com/projects/Aphelida_Extended_Data/111539, on 17 March 2023. Green and blue background indicates the *A. insulamus* and *A. tribonematis* sequences, respectively, used for phylogenetic analysis (Figure 6). Bold letters indicate the p25alpha domains; red letters indicate the LxxxFxxFxxF and GxGxGxxGR conservative sequences.
